# Dermal Filler Use in Patients Undergoing Chemotherapy and Radiation Therapy

**DOI:** 10.2196/76898

**Published:** 2025-09-09

**Authors:** Mia Panlilio, Elizabeth Tchernogorova, Binh Minh Nguyen, Sana Khan, Leslie Torgerson

**Affiliations:** 1 College of Osteopathic Medicine, Rocky Vista University, 8401 S Chambers Road, Parker, CO, 80112, United States, 1 9253236431; 2 College of Osteopathic Medicine, Rocky Vista University, Ivins, UT, United States

**Keywords:** dermal, filler, chemotherapy, cosmetic, aesthetic, radiation

## Abstract

Dermal fillers have gained increasing popularity for their ability to enhance facial symmetry, restore volume, and improve skin texture. However, their use in patients with cancer undergoing active chemotherapy and radiation therapy poses unique challenges, as these treatments can alter both the safety profile and efficacy of filler procedures. Chemotherapy can interfere with normal wound healing and immune responses, warranting a more cautious and individualized approach when considering dermal fillers in this population. Although rare, dermal fillers have been associated with adverse outcomes in a limited number of diseases, including cellulitis, autoimmune/inflammatory syndrome induced by adjuvants, and a possible predisposition to malignancy. Other effects include localized inflammatory, systemic hypersensitivity, and delayed granulomatous formation, and these could be more severe in patients undergoing antineoplastic therapy. Furthermore, chemotherapy is often paired with adjuvant radiation therapy in cancer treatment, making it important to note the potential changes radiation can have on the skin. More research is needed to examine the direct interactions of chemotherapy and radiation on various filler materials injected within the skin, and how these can alter one’s risk of adverse effects. The lack of research on this topic further emphasizes that clinicians should thoroughly educate patients receiving chemotherapy and adjuvant radiation treatment about the heightened potential risks associated with dermal filler injections and treatment regimens should be planned accordingly to minimize any adverse events.

## Introduction

Dermal fillers, which are minimally invasive cosmetic treatments, have gained widespread popularity for their ability to restore volume, soften wrinkles, and enhance facial contours. Various filler types are available, including hyaluronic acid-based products, calcium hydroxylapatite, poly-L-lactic acid, polymethyl methacrylate, and silicone, each offering unique properties and applications [1,2].

Hyaluronic acid-based fillers are one of the most popular types that are used for fine lines and wrinkles, lasting about 6 to 12 months before naturally getting absorbed by the body [1]. Calcium hydroxylapatite fillers are a thicker consistency product used for volume restoration in deeper lines and wrinkles, and they typically last 12 months or longer [1]. Poly-L-lactic acid fillers are also popular, and are used to treat deeper facial wrinkles, with results lasting more than 2 years [1]. Polymethyl methacrylate is a physical filler that comes in the form of a microsphere, or tiny ball, that remains under the skin indefinitely for long term support, and it also contains collagen which provides structure and firmness [1]. Silicone fillers have been used in some parts of the world for volume restoration; however, silicone filler products currently are not approved by the Food and Drug Administration (FDA) in the United States and have fallen out of favor due to the high occurrence of filler migration, swelling and tissue damage, and severe inflammatory reactions to the material [1].

The rising use of fillers can be attributed to their effectiveness, convenience, and the growing demand for non-surgical aesthetic enhancements. However, these treatments are not without risks. Contraindications include allergies, infections, and certain medical conditions that could heighten the risk of infection or foreign body reactions, such as immunosuppressive or autoimmune conditions. Of particular concern is the use of dermal fillers in patients actively undergoing chemotherapy and radiation therapy. Chemotherapy and radiation can compromise immune function and tissue repair mechanisms, potentially altering both the safety and effectiveness of filler procedures. These therapies may disrupt normal wound healing and immune surveillance, necessitating a more cautious and individualized approach when considering dermal filler use in oncology patients. This viewpoint aims to evaluate the current literature on the safety and potential adverse effects of dermal filler use in patients undergoing chemotherapy and radiation therapy.

## Adverse Effects of Fillers

With the increase in popularity and accessibility of aesthetic treatments, there has been significant growth in the research sector. In 2024, the United States ranked #1 for the most published articles on soft tissue filler injections globally [3]. However, there remains a significant lack of research for the effects of dermal filler injections on patients undergoing chemotherapy and radiation therapy. Dermal fillers can cause adverse effects such as localized inflammatory, systemic hypersensitivity, delayed granulomatous formation, cellulitis, and autoimmune/inflammatory syndrome induced by adjuvants (ASIA) [4-9]. Additionally, while generally considered safe, dermal fillers, particularly those composed of hyaluronic acid (HA), introduce exogenous compounds into the tissue environment [10]. Endogenous HA is highly concentrated in some malignant neoplasms and its interaction with the CD44 receptor is implicated in tumor growth and poor prognosis [10]. While there is theoretical concern that exogenous HA fragments may contribute to a pro-tumor microenvironment, direct evidence linking fillers to new malignancy is lacking.

## Case Reports in Oncology Patients

While there has been minimal research conducted on the topic of dermal filler reactions related to chemotherapy and radiation therapy, there are a few case studies describing reactions of dermal fillers in relation to these treatments, as summarized in Table 1.

One case report was about a 53-year-old woman with advanced non-small cell lung cancer who received carboxymethyl cellulose and polycaprolactone microsphere injection in her anterior neck 15 months previously [11]. She developed nodules and hardened folds along her sternocleidomastoid muscle bilaterally while receiving a course of 5 nivolumab infusions [11]. These nodules and hardened folds were later determined to be foreign body reactions to the filler. The patient also developed autoimmune colitis during the nivolumab infusions and was administered systemic glucocorticoids, which resolved both the autoimmune colitis and foreign body reaction to the filler. This case exemplifies how patients with preexisting fillers have an increased risk of developing a foreign body reaction when initiating chemotherapy. Additionally, the administration of glucocorticoids and subsequent resolution of the foreign body reaction provides some support towards using glucocorticoids to treat these reactions in this patient population.

Another case report was about a woman in her 60s who received dermal filler 25 years prior to initiating chemotherapy; she developed painless facial nodules after 2 courses of ipilimumab treatment [12]. The nodules were excised and later confirmed to be a foreign body reaction to filler via histology. Like the previous case, this again highlights the increased risk of foreign body reactions in patients with preexisting fillers who are undergoing chemotherapy and shows the risk is still heightened years after filler injection.

In a case report published in 2019, a 52-year-old man with preexisting HA fillers in his cheeks, undergoing cetuximab and radiation for glossotonsillary malignancy, developed significant inflammation and edema at the filler sites within hours of his first cetuximab dose [5]. This acute reaction was distinct from typical cetuximab-induced rashes and subsided rapidly after the fillers were dissolved. This exemplifies a more atypical and rapid presentation of a chemotherapy-induced foreign body reaction to fillers and highlights how filler dissolution could be used to resolve the reaction.

**Table 1.** Summary of the presented case reports.

Patient characteristics	Filler type	Cancer therapy	Time between filler injection and reaction	Adverse events	Outcomes
53-year-old woman with NSCLC^a^	Carboxymethyl cellulose and polycaprolactone microsphere	Nivolumab	15 months	Bilateral nodules and indurated folds along the sternocleidomastoid muscles, later found to be a foreign body reaction to the filler	Patient also developed autoimmune colitis from nivolumab treatment and was treated with systemic glucocorticoids, which also resolved the filler foreign body reaction
61-year-old woman with superficial spreading melanoma in the left scapular region	Unspecified synthetic filler material	Ipilimumab	25 years	Painless granulomatous facial nodules found to be a foreign body reaction to the filler	All granulomatous nodules were excised
52-year-old man with glossotonsillary malignancy	Hyaluronic acid	Cetuximab and radiation therapy	2 hours	Inflammation and edema around filler sites	Acute reaction rapidly resolved following filler dissolution
43-year-old woman with chronic myeloid leukemia	Hyaluronic acid	Imatinib mesylate	Filler injected during chemotherapy treatment	None mentioned	Successful treatment

a NSCLC: non-small cell lung cancer.

One case report in Korea described “successful” HA filler injection in a patient actively undergoing imatinib mesylate treatment for chronic myeloid leukemia and was presented in 2019 [13]. However, researchers only followed the patient for 10 weeks after filler treatment, so possible reactions occurring after 10 weeks were not described. The lack of longer-term follow-up therefore makes this case a questionable example of a truly “successful” filler treatment.

Other case studies available mostly describe foreign body reactions occurring after longer time periods, such as the case report describing a reaction 25 years after dermal filler injection [12]. Additionally, there are no studies specifying filler reactions with certain types of chemotherapy agents. For example, imatinib mesylate is a tyrosine kinase inhibitor, whereas ipilimumab is a CTLA-4 inhibitor. The differences in each drug’s mechanism of action could impact the type of a reaction a patient experiences based on the immunological pathways affected. This makes it difficult for physicians and scientists to determine the true long-term impacts and efficacy of dermal filler on patients undergoing chemotherapy treatment without further research on reactions between specific filler materials and chemotherapies of different drug classes.

## Effects of Radiation on the Skin

In a prospective cohort study done in 2014, the effects of radiotherapy were studied to see what changes occur within the skin when radiation is used for the treatment of breast cancer [14]. After radiotherapy, the irradiated breast showed a notable decrease in skin hydration, an increase in skin pH, increase in pigmentation, and increase in cutaneous blood flow. Radiotherapy is also known to damage skin barrier function because it induces apoptosis and necrosis of epidermal cells, thus decreasing the production of natural moisturizing factors and intercellular lipids [6]. It also causes an alkalinization of the stratum corneum, which is a layer of the skin that favors bacterial and fungal proliferation, thus increasing the risk of infection [14]. When it comes to possible interactions radiation therapy can have in patients with existing fillers, there are no current clinical studies available.

## Specific Patient Risks

Patients undergoing chemotherapy or those who are immunosuppressed face unique challenges when considering dermal fillers. Chemotherapy can significantly impact the immune system, increasing the risk of infections and other adverse effects. The American Society of Clinical Oncology (ASCO) and the Infectious Diseases Society of America (IDSA) highlight that patients with cancer-related immunosuppression are particularly vulnerable to infections due to neutropenia and other factors [15]. Neutropenia, a common side effect of chemotherapy, reduces the body’s ability to fight infections, making patients more susceptible to bacterial and fungal infections. This increased susceptibility can lead to adverse effects such as cellulitis, delayed wound healing, and atypical inflammatory reactions following dermal filler injections [15].

A study presented at the 2024 ASCO Annual Meeting assessed the safety and quality of life in oncology patients receiving dermal fillers during active cancer therapy. The study found that fillers can improve quality of life and aesthetic outcomes, such as improving measured quality of life scores, making patients appear less ill, sad, or distracted, and about 81% of patients in the study reported feeling satisfied following injections and would plan future aesthetic treatments [4]. Despite these reported improvements, there were minor dermal side effects in 13 out of the 127 patients included in the study, and 1 patient who developed a delayed inflammatory reaction [4]. Additionally, a systematic review noted that immunosuppressed patients, including those on chemotherapy, may have an increased risk of adverse effects such as filler granulomas and infectious complications [1,9].

## Recommendations for Clinicians

Chemotherapy and radiation can cause tissue volume loss, skin dryness, sensitivity, and changes in pigmentation [2,6]. Cosmetic treatments such as dermal fillers can help patients with cancer feel more comfortable with their appearance, boosting self-esteem, contributing to a sense of increased psychological well-being and improving their quality of life [2]. Clinicians should therefore acknowledge and proactively address these crucial psychosocial benefits. However, this presents a dual consideration: while offering aesthetic and psychological advantages, these interventions simultaneously carry an elevated risk of adverse effects within patient populations undergoing chemotherapy and radiation therapies. Therefore, clinicians must thoroughly educate patients about these potential interactions and complications.

Consultations regarding dermal filler treatment in this specific patient population calls for a discussion involving the patient’s oncologist, dermatologist, and injector. This multi-disciplinary discussion is crucial to obtain a comprehensive analysis of the patient to determine the safest and most effective cosmetic plan. To guide this process, a pre-treatment checklist should include (1) a review of the patient’s cancer treatment timeline, (2) analysis of the patient’s immune status or susceptibility to infections, (3) assessment of skin integrity (especially in irradiated areas), (4) documentation of any prior filler use, and (5) a clear discussion of risks, benefits, and procedure timing relative to immunosuppressive therapy.

The treating provider should also emphasize the importance of using aseptic techniques during the procedure and closely monitor patients post-injection to promptly manage any adverse reactions.

## Conclusion

When considering dermal filler use in patients undergoing chemotherapy and radiation therapy, a tailored, risk-informed approach is essential. An in-depth consultation between the patient, the patient’s oncologist, and their injector should involve a thorough analysis of the patient’s skin, as well as answering questions regarding what kind of cancer treatment they are receiving, and how recent was their last treatment with chemotherapy or radiation. Given the altered immune function and changes to the skin’s structural integrity caused by chemotherapy and radiation, this population faces an elevated risk of infection, delayed wound healing, and immune-mediated filler complications. While the therapeutic benefits of cosmetic restoration, such as improved self-image and quality of life, are meaningful, they must be balanced against these unique risks.

Currently, there is minimal research that has been done to show exactly what adverse effects can occur from dermal filler treatments in patients who are actively undergoing chemotherapy or radiation therapy. Furthermore, the included case reports vary in their level of clinical detail, histological confirmation, follow-up duration, use of fillers, and their combinations with cancer therapies. This heterogeneity limits the ability to compare cases systematically or draw consistent conclusions. Some papers fail to specify the exact type, brand, or batch of filler used, which hinders the ability to assess biocompatibility or degradation in immunocompromised hosts. The proposed recommendations in this viewpoint are extrapolated from what is currently known about how chemotherapy and radiation therapy affect the skin, and the adverse effects of dermal filler treatment. The first filler agent approved by the FDA for cosmetics was Zyderm in 1981 [16]. There have been hundreds of new agents developed between 1981 and 2025, and research on long-term effects of varying fillers are developing with time [16].

More research is needed to examine the direct interactions of chemotherapy and radiation on various filler materials injected within the skin, and how these interactions can increase or decrease the risk of adverse effects. Future investigation should prioritize comparative studies that analyze risks among patients receiving different classes of chemotherapies (eg, immune checkpoint inhibitors vs tyrosine kinase inhibitors) and explore whether treatment timing (active therapy or remission) affects the likelihood of adverse filler-related events. Additional multi-center, prospective studies should also be done to address the gaps identified.

## Abbreviations

ASCO: American Society of Clinical Oncology
ASIA: autoimmune/inflammatory syndrome induced by adjuvants
FDA: Food and Drug Administration
HA: hyaluronic acid
IDSA: Infectious Diseases Society of America

## References

[1] Guo J, Fang W, Wang F (2023). Injectable fillers: current status, physicochemical properties, function mechanism, and perspectives. RSC Adv.

[2] Tejero P, Pinto H (2020). Aesthetic Treatments for the Oncology Patient.

[3] Liao ZF, Cong LY, Li FW (2024). The research trend of soft tissue filler injection from 2000 to 2022: a bibliometric and visualized analysis. Plast Reconstr Surg Glob Open.

[4] Shachar E, Hasson E, Arzi O (2024). Aesthetics treatment with injectable fillers in patients with cancer: safety and quality of life during active antineoplastic therapy. JCO.

[5] Pathmanathan S, Dzienis M (2019). Cetuximab associated dermal filler reaction. BMJ Case Rep.

[6] Proietti I, Skroza N, Mambrin A (2021). Aesthetic treatments in cancer patients. Clin Cosmet Investig Dermatol.

[7] Owczarczyk-Saczonek A, De Boulle K (2023). Hyaluronic acid fillers and ASIA syndrome: case studies. Clin Cosmet Investig Dermatol.

[8] Heydenrych I, De Boulle K, Kapoor KM, Bertossi D (2021). The 10-point plan 2021: updated concepts for improved procedural safety during facial filler treatments. Clin Cosmet Investig Dermatol.

[9] Edwards PC, Fantasia JE (2007). Review of long-term adverse effects associated with the use of chemically-modified animal and nonanimal source hyaluronic acid dermal fillers. Clin Interv Aging.

[10] Hill A, McFarlane S, Johnston PG, Waugh DJJ (2006). The emerging role of CD44 in regulating skeletal micrometastasis. Cancer Lett.

[11] Syunyaeva Z, Kahnert K, Kauffmann-Guerrero D, Huber RM, Tufman A (2018). Dermal filler injections mimic tumor activity during immune checkpoint inhibition. Respiration.

[12] Bisschop C, Bruijn MS, Stenekes MW, Diercks GFH, Hospers GAP (2016). Foreign body reaction triggered by cytotoxic T lymphocyte-associated protein 4 blockade 25 years after dermal filler injection. Br J Dermatol.

[13] Sung Y, Kim MH, Cho E (2019). Successful hyaluronic acid filler injection in a chronic myeloid leukemia patient taking imatinib mesylate. J Cosmet Laser Ther.

[14] Hu SCS, Hou MF, Luo KH (2014). Changes in biophysical properties of the skin following radiotherapy for breast cancer. J Dermatol.

[15] Taplitz RA, Kennedy EB, Bow EJ (2018). Antimicrobial prophylaxis for adult patients with cancer-related immunosuppression: ASCO and IDSA clinical practice guideline update. J Clin Oncol.

[16] Matton G, Anseeuw A, De Keyser F (1985). The history of injectable biomaterials and the biology of collagen. Aesthetic Plast Surg.

